# Mutual Promotion of *LAP2* and *CAT2* Synergistically Regulates Plant Salt and Osmotic Stress Tolerance

**DOI:** 10.3389/fpls.2021.672672

**Published:** 2021-06-09

**Authors:** Yu Zhang, Lin-Feng Wang, Ting-Ting Li, Wen-Cheng Liu

**Affiliations:** ^1^State Key Laboratory of Crop Stress Adaptation and Improvement, School of Life Sciences, Henan University, Kaifeng, China; ^2^State Key Laboratory of Cotton Biology, School of Life Sciences, Henan University, Kaifeng, China; ^3^Jiangsu Key Laboratory of Marine Pharmaceutical Compound Screening, Jiangsu Ocean University, Lianyungang, China

**Keywords:** H_2_O_2_, *CAT2*, *LAP2*, oxidative stress, interaction

## Abstract

Almost all abiotic stresses induce reactive oxygen species (ROS) overaccumulation, causing oxidative damages to plant cells. Catalase (CAT) plays a vital role in plant oxidative stress tolerance by scavenging stress-induced excess H_2_O_2_; thus, the identification of factors regulating catalase function will shed light on the underlying regulatory mechanisms. Here, we identified leucine aminopeptidase 2 (*LAP2*) as a novel *CAT2*-interacting protein and showed a mutual promotion effect of the two proteins in plant stress responses. *LAP2* has a physical interaction with *CAT2* in plant cells. The loss-of-function mutant of *LAP2*, *lap2-3*, is hypersensitive to salt or osmotic stress with increased ROS accumulation and malondialdehyde content and decreased catalase activity. The *lap2-3* mutant has less *CAT2* protein levels as *CAT2* protein stability is impaired in the mutant. Scavenging excess ROS by glutathione or overexpressing *CAT2* in the *lap2-3* mutant recovers its hypersensitive phenotype to salt or osmotic stress. Further study showed that *CAT2* promotes *LAP2* hydrolysis activity with leucine-4-methylcoumaryl-7-amides as a substrate *in vivo* and *in vitro*, and thus, similar to the *lap2-3* mutant, the *cat2-1* mutant also has lower γ-aminobutyric acid content than the wild type. Together, our study reveals mutual promotion effects of *CAT2* and *LAP2* in conferring plant salt and osmotic stress tolerance.

## Introduction

Soil salinity and water deficit are abiotic stresses that severely affect crop growth, quantity, and quality, causing enormous economic losses and serious food insecurity worldwide (Yang and Guo, 2018; Yu et al., 2020). Due to their sessile lifestyle, plants usually cannot avoid high salinity- or drought-induced injuries by directly changing their location; thus, plants have developed sophisticated adaptive mechanisms to respond to these stresses so as to survive and reproduce themselves (Li et al., 2015). Both salt and drought stresses reduce plant water uptake and disturb plant normal physiological processes, bringing about not only ionic and osmotic stresses but also oxidative damages which are secondary effects resulting from stress-induced overaccumulation of reactive oxygen species (ROS) including hydroxyl radicals, hydrogen peroxide (H_2_O_2_), and superoxide anions (Liu and He, 2016; Waszczak et al., 2018; Yang and Guo, 2018). The overaccumulated ROS can damage plant biomacromolecules such as protein, DNA, and lipids, causing protein dysfunction, DNA strand breakage, and lipid peroxidation (Umeno et al., 2017; Yuan et al., 2017). Thus, reducing stress-induced ROS overaccumulation is one of the most important and common protective mechanisms for plants under an adverse environment.

Reactive oxygen species-scavenging enzymes including superoxide dismutase (SOD), catalase (CAT), peroxidase, peroxiredoxin, and non-enzymatic small molecules contribute to maintain ROS homeostasis by removing stress-induced excess ROS in plant cells (Hu et al., 2010; Sanchez-McSweeney et al., 2021). Among these ROS-scavenging enzymes, CAT plays a vital role in plant tolerance to high salinity and drought. Early reports showed that mutation of *CAT2* results in increased H_2_O_2_ accumulation and decreased tolerance to salt stress in *Arabidopsis* plants (Bueso et al., 2007). Similarly, a loss-of-function mutant of *CAT3* with higher H_2_O_2_ accumulation in the leaves is severely sensitive to water deficit, while its overexpression transgenic plants are more tolerant than the wild-type plants when challenged with drought stress (Zou et al., 2015).

To respond to salt or drought stress, plants upregulate the expression of *CATs* to repress stress-induced ROS accumulation and reduce oxidative damages (Luna et al., 2005; Sharma and Dubey, 2005; Xing et al., 2007; Gondim et al., 2012; Wei et al., 2020). It is reported that a MAP kinase, MEK1, positively regulates the expression of all three *Arabidopsis CATs* (*CAT1*, *CAT2*, and *CAT3*) (Xing et al., 2007). Thus, compared with the wild-type plants, the *mek1* mutant is more sensitive, but MEK1-overexpressing transgenic lines are more tolerant to both salt and drought stresses with lower or higher stress-induced *CAT* expression, respectively (Xing et al., 2007). In addition to the transcriptional regulation, catalase activity is also regulated by interacting partners in the plant’s response to salt or drought stress. For example, Calcium-dependent Protein Kinases8 (CPK8) interacts with and phosphorylates CAT3, stimulating its catalase activity and thus enhancing drought stress tolerance in *Arabidopsis* (Zou et al., 2015). Also, the protein NO CATALASE ACTIVITY1 (NCA1), as a chaperone of *CAT2*, confers *Arabidopsis* plants increased salt stress tolerance possibly through maintaining the folding of *CAT2* protein in a functional state (Li et al., 2015). In addition, a peroxisome-located small heat shock protein, Hsp17.6CII, interacts with and activates catalases, conferring *Arabidopsis* plants alkaline and salt stress (Li et al., 2017). A recent report documented that Heat Shock Protein90.9 (HSP90.9) interacts with and promotes Catalase1 in *Manihot esculenta*; thus, *MeHSP90.9*-silenced plants are more sensitive to drought stress with lower catalase activity and higher H_2_O_2_ accumulation (Wei et al., 2020). These reports reveal that catalase-interacting proteins play a vital role in regulating catalase activity and plant abiotic stress tolerance, and thus, the identification of more CAT-interacting proteins would provide more insights into the mechanisms underlying the regulation of oxidative stress tolerance in plant’s response to salt or drought stress.

Peptidases, ubiquitous proteins in all living organisms, play fundamental roles in intracellular protein turnover and have important functions in plant growth and response to exogenous environmental changes (Waditee-Sirisattha et al., 2011). Peptidases have hydrolysis activity of liberating amino acids from the N-terminal end of proteins or peptides (Pautot et al., 2001). Leucine aminopeptidase 2 (*LAP2*), as a member of the M17 peptidase gene family, affects a variety of plant growth processes and stress sensitivity including high salinity and drought through changes of gene expression, amino acids, and γ-aminobutyric acid (GABA) (Waditee-Sirisattha et al., 2011). *Arabidopsis LAP2* and its homologous proteins in tomato (LAP-A and LAP-N) also act as molecular chaperones to alleviate stress-induced damages to target proteins (Scranton et al., 2012). These reports clearly showed an important role of *LAP2* in plant stress tolerance; however, the underlying mechanism remains elusive.

In this study, we identified *LAP2* as a new *CAT2*-interacting partner and revealed that, on the one hand, *LAP2* increases plant salt and drought stress tolerance by interacting with and stabilizing *CAT2* protein to reduce ROS accumulation; on the other hand, *CAT2* also stimulates *LAP2* hydrolysis activity to increase GABA content. Our study reveals a mutual promotion effect of *LAP2* and *CAT2* on plant salt and osmotic stress tolerance.

## Materials and Methods

### Plant Material and Growth Conditions

*Arabidopsis thaliana* ecotype Columbia was used in this study. The knockout mutant of *CAT2* (*cat2-1*, SALK_076998) was previously reported (Zhang et al., 2020b), and the *lap2-3* mutant (SAIL_192_A08) was obtained from the Arabidopsis Biological Resource Center (ABRC). Seeds were surface sterilized for 5 min in 5% commercial bleach, washed three times with sterile water, and plated on half-strength Murashige and Skoog (MS) medium (pH 5.8) (Sigma-Aldrich) containing 1% sucrose and 1% agar. Plants were stratified at 4°C for 3 days in the dark, and then transferred to chambers. The seedlings grown vertically at 22°C and 100 μmol m^–2^ s^–1^ illumination under 16 h light/8 h dark conditions for 5 days were transferred to half strength MS medium without or with 125 mM NaCl or 250 mM mannitol (Sigma-Aldrich) for another 5 days, and then the fresh weight was measured.

For germination assays, *Arabidopsis* seeds were sown on half-strength MS medium with 125 mM NaCl or 250 mM mannitol (Sigma-Aldrich), kept in the dark at 4°C for 3 days, and transferred to chambers for 5 days, and then the percentage of green cotyledons was calculated.

For glutathione (GSH) treatment, GSH was dissolved in ddH_2_O to make a 100-mM stock solution and frozen at –20°C until used. When the autoclaved agar medium was cooled to below 50°C, the freshly prepared stock solution was directly added to the 1/2 MS medium to a final concentration of 100 μM, and then the medium was poured immediately into petri dishes and used to treat plant seedlings.

For cycloheximide (CHX) treatment, 5-days-old wild-type and *lap2-3* mutant seedlings grown on 1/2 MS medium were transferred to liquid 1/2 MS medium containing 50 μM CHX and treated for 0, 2, 4, or 6 h. The treated seedlings were then harvested for immunoblot analysis of *CAT2* protein accumulation using anti-*CAT2* antibody.

### 3,3-Diaminobenzidine Staining and Nitroblue Tetrazolium Staining

Five-days-old seedlings were transferred to half-strength MS medium without or with 125 mM NaCl or 250 mM mannitol (Sigma-Aldrich) for 5 days, and then used for 3,3-diaminobenzidine (DAB) or nitroblue tetrazolium (NBT) staining to assay H_2_O_2_ or superoxide anion accumulation as described previously (Guan et al., 2013; Zhang et al., 2020b,c). For DAB staining, the seedlings were incubated in freshly prepared DAB staining solution (1 mg/ml DAB and 0.1% Tween 20 in 10 mM Na_2_HPO_4_) for 8 h, and then rinsed with 70% ethanol for several times to remove the chlorophyll. The images of the leaves were captured using a digital camera. For superoxide anion staining, the seedlings were vacuum infiltrated with 0.1 mg/ml NBT in 25 mM HEPES buffer (pH 7.6) for 2 h in darkness. Chlorophyll was removed by using 70% ethanol, and then the images of the leaves were captured using a digital camera. For DAB or NBT staining intensity quantification, images were first transformed into 8-bit type images and inverted, and the mean gray values of the image background and seedling leaves were collected using Photoshop CS5 (Adobe). The gray values were used to analyze the relative intensity of DAB staining or NBT staining. Three biological replicates were performed (a group of 10 seedlings was used as one biological sample), and thus, 30 seedlings were employed for the DAB or NBT staining intensity analysis.

### Measurement of Malondialdehyde Content

Malondialdehyde (MDA) content was measured according to a previously described method (Zhang et al., 2020a). Briefly, 0.2 g of treated or untreated seedlings were ground in 1 ml of chilled reagent (0.25% thiobarbituric acid in 10% trichloroacetic acid). After incubation at 100°C for 30 min, the extracts were cooled at room temperature and centrifuged at 12,000*g* for 15 min. The absorbance of the supernatant was measured at 532, 450, and 600 nm. The MDA content was calculated based on the following equation: 6.45 × (OD_532_ – OD_600_) – 0.559 × OD_450_.

### Detection of Catalase Activity

Measurement of catalase activity was performed according to our previous report (Zhang et al., 2020b). Briefly, treated or untreated seedlings were ground with liquid nitrogen and then resuspended in cold extraction buffer (50 mM *Tris*-HCl, pH 7.4 and 10% glycerol). After centrifugation at 12,000*g* at 4°C for 15 min, the supernatant was used for assaying catalase activity using a Catalase Assay Kit (Beyotime Biotechnology) according to the manufacturer’s instructions.

### Generation of Complementation (Com) Lines of the *lap2-3* Mutant

The genomic sequence of 1,400 bp upstream of *LAP2* translation start codon (ATG) and the full-length coding sequence (CDS) of *LAP2* were amplified and cloned into pCAMBIA1300 between the *Eco*RI/*Kpn*I and *Sac*I/*Pst*I restriction sites, respectively. The resulting plasmid was introduced into the *lap2-3* mutant *via Agrobacterium tumefaciens*-mediated floral transformation. Com T4 transgenic plants were used for phenotypic analysis.

### Plasmid Construction and Plant Transformation

The full-length coding sequence of *CAT2* was amplified using PCR and cloned into pEGAD, resulting in 35S:GFP-*CAT2*, and then introduced into the *lap2-3* mutant using an *A. tumefaciens* (GV3101)-mediated transformation and the floral dip method. The T4 transgenic plants were used for phenotypic analysis.

### Quantitative Real-Time PCR

Treated or untreated seedlings were collected for total RNA isolation, first-strand cDNA synthesis, and qRT-PCR as we described previously (Liu et al., 2018). The constitutively expressed *ACTIN2/8* gene was used as an internal control. All experiments were repeated at least three times. The primer sequences are listed in Suppmentary Table 1.

### Yeast Two-Hybrid Assay

To assay the interaction between *CAT2* and *LAP2* in yeast, the full-length CDS of *LAP2* was cloned into pGADT7, and the plasmid pGBKT7–*CAT2* was previously reported (Yuan et al., 2017). The yeast transformation and growth were carried out with the Matchmaker system (Clontech). Primer sequences are listed in Suppmentary Table 1.

### Luciferase Complementation Imaging Assays

The CDS of *CAT2* and *LAP2* were in cloned into the JW772 and JW771 vectors containing n-LUC and c-LUC, respectively. The resultant plasmids were transformed into *A. tumefaciens* (C58C1) and used to infiltrate *Nicotiana benthamiana* leaves. Three days after infiltration, the leaves were incubated in PBS solution containing 150 μg/ml D-luciferin potassium salt in the dark for 10 min before luminescence assay. The luciferase (LUC) image was captured by NightSHADE LB 985 according to the manufacturer’s instructions.

### Co-immunoprecipitation Assays

To assay the interaction between *CAT2* and *LAP2* in plants, the CDS of *CAT2* was amplified using PCR with specific primers containing Flag sequence (GATTACAAGGATGACGACGATAAG), and then inserted into the pBARN vector at *Sma*I site behind the 35S promoter, resulting in 35S:*CAT2*-Flag. The CDS of *LAP2* was cloned into the pEGAD vector at *Bam*HI site, resulting in 35S:GFP–*LAP2*. The resultant plasmids were transformed into *Agrobacterium* GV3101 and used to infiltrate *N. benthamiana* leaves. Three days after infiltration, total proteins were extracted from the leaves and immunoprecipitated with gel-conjugated mouse anti-GFP mAb (ABclonal, #AE064, dilution at 1:1,000). The precipitants were resuspended, separated by SDS-PAGE gel, and then immunoblotted with anti-Flag.

### Bimolecular Fluorescence Complementation Assays

For the bimolecular fluorescence complementation (BiFC) assays, the CDS of *Arabidopsis CAT2* and *LAP2* were cloned into pSP-YNE and pSP-YCE, respectively, resulting in 35S:*CAT2*-YFPn and 35S:*LAP2*-YFPc. The resultant plasmids were introduced into *A. tumefaciens* (GV3101) and used to infiltrate *N. benthamiana* leaves. Three days after infiltration, the leaves were used to observe the reconstituted YFP fluorescent signal under a confocal laser scanning microscope (Carl Zeiss LSM710 META laser scanning microscope).

### Protein Expression and Purification

The pET28a-*CAT2* plasmid and His-tagged *CAT2* protein expression and purification were previously reported (Yuan et al., 2017). The CDS of *LAP2* was cloned into pGEX4T-1 and transformed into *Escherichia coli* BL21 (DE3). The GST-fused *LAP2* protein was expressed with 0.5 mM isopropyl β-D-thiogalactopyranoside at 22°C for 12 h and then purified using GST agarose (Thermo Scientific) according to the manufacturer’s instructions.

### Detection of *LAP2* Activity

The *LAP2* activity with the synthetic fluorogenic substrate leucine-4-methylcoumaryl-7-amides (Leu-MCA) was performed according to a previously reported method (Waditee-Sirisattha et al., 2011). Briefly, the reaction mixture (100 μM of Leu-MCA, 50 mM *Tris-*Cl buffer, pH 8.5, 2.0 mM MnSO_4_) was incubated at 37°C. Once the purified *LAP2* or *CAT2* protein was added, the fluorescence signal of 7-amino-4-methylcoumarin released by *LAP2* hydrolysis activity was measured at an excitation wavelength of 360 nm and an emission wavelength of 460 nm by BioTek Cytation 3 for 45 min. According to a previous report, the fluorescence intensities at 30 min were further analyzed (Waditee-Sirisattha et al., 2011). Three independent biological replicates were performed.

### Measurement of GABA Content

Detection of GABA content in the wild-type and mutant seedlings was modified from previously reported methods (Liu et al., 2018; Yue et al., 2018). Briefly, 0.1 g of the wild-type *lap2-3* and *cat2-1* mutant seedlings were ground with liquid nitrogen to a fine powder and extracted using 1 ml acetonitrile. After centrifugation at 12,000*g* for 15 min, the supernatant was transferred and dried under nitrogen gas in a new tube. The crude extract resuspended in 100 μl acetonitrile containing 1% 2,4-dinitrofuorobenzene was mixed with 100 μl of 0.1 M Na_2_B_4_O_7_ and then incubated at 60°C for 1 h in the dark. After derivatization, GABA standards and the samples were, respectively, separated and monitored by Agilent 1100 HPLC (C18 reversed-phase column) with the mobile phase (acetonitrile and phosphate buffer) at a flow rate of 1.0 ml/min at 360 nm.

### Statistical Analysis

Data are means (±SD) of three biological replicates, and the asterisks indicate statistically significant differences (^∗^*p* < 0.05, ^∗∗^*p* < 0.01, and ^∗∗∗^*p* < 0.001, Student’s *t* test). Bars with different letters indicate significant differences at *p* < 0.05 by two-way ANOVA with Tukey’s multiple comparison test.

## Results

### Leucine Aminopeptidase2 Interacts With *CAT2*

**FIGURE 1:**
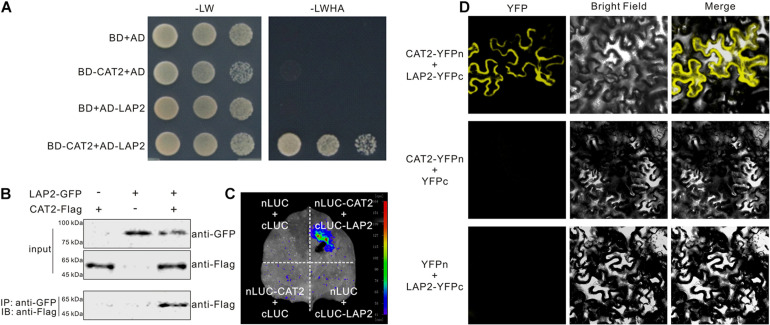
*CAT2* interacts with leucine aminopeptidase 2 (*LAP2*). **(A)** The interaction between *CAT2* and *LAP2* was examined by yeast two-hybrid experiment. **(B)** Co-IP assays showing *CAT2*–*LAP2* interaction. 35S:*LAP2*-GFP and 35S:*CAT2*-Flag were co-expressed in *Nicotiana benthamiana* leaves for 3 days *via Agrobacterium*-mediated transformation. Total proteins were extracted and immunoprecipitated with anti-GFP antibody, and the precipitants were then immunoblotted with anti-Flag. **(C)** *CAT2* interacts with *LAP2* in *N. benthamiana* leaves by the LCI assay. **(D)** *CAT2* interacts with *LAP2* in *N. benthamiana* leaves as indicated by BiFC assays.

*CAT2* plays a vital role in plant salt and drought stress tolerance by scavenging stress-induced H_2_O_2_; thus, identification of factors regulating *CAT2* function will shed light on the mechanisms underlying plant responses to high salinity or drought-induced oxidative stress. In our previous report showing that *CAT2* functions in plant resistance to the necrotrophic pathogen *Botrytis cinerea* infection (Yuan et al., 2017), we also identified *LAP2* as a *CAT2*-interacting protein, but we did not show the screening result previously. We therefore cloned the full-length coding sequence of *LAP2* and performed a yeast two-hybrid experiment to examine the interaction between *CAT2* and *LAP2*. Our result showed that *LAP2* interacts with *CAT2* in yeast (Figure 1A). The interaction between *CAT2* and *LAP2* was confirmed by co-immunoprecipitation (co-IP) experiment. GFP-tagged *LAP2* and Flag-tagged *CAT2* were transiently co-expressed in *N. benthamiana* leaves, and *CAT2*-Flag could be immunoprecipitated using anti-GFP antibody, indicating that *CAT2* and *LAP2* form a complex in plant cells (Figure 1B). Also, the *CAT2*–*LAP2* interaction was verified using a LUC complementation imaging assay in which nLUC-*CAT2* and cLUC-*LAP2* were co-expressed in *N. benthamiana* leaves. Luciferase fluorescence could be detected in *N. benthamiana* leaves after incubated with the substrate of luciferase–luciferin (Figure 1C). Additionally, we also examined the interaction of the two proteins with BiFC assays by expressing YFPn-tagged *CAT2* and YFPc-tagged *LAP2*. Our confocal microscopy results showed that a strong florescence signal was detected in the cytosol (Figure 1D), suggesting that *LAP2* interacts with *CAT2* in the cytosol, similar with the previously reported localization of *CAT2*–NAC1 interaction or *CAT2*–CPK3 interaction (Li et al., 2015).

### Leucine Aminopeptidase2 Is Required for Plant Stress Tolerance by Affecting ROS Accumulation and Catalase Activity

**FIGURE 2:**
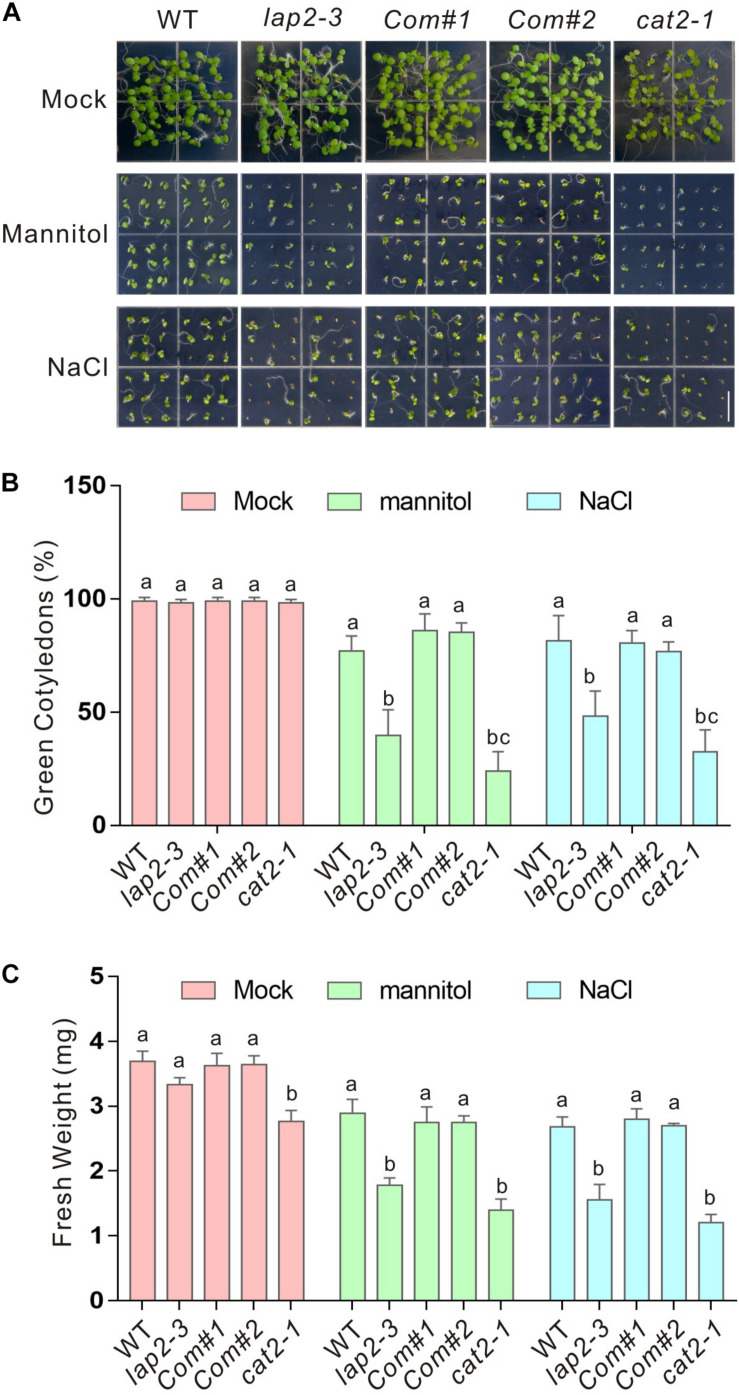
The *lap2-3* mutant is sensitive to salt or osmotic stress. **(A)** Images of 5-days-old wild-type, *lap2-3*, *LAP2* complementation lines and *cat2-1* mutant seeds germinated on 1/2 MS medium without or with 250 mM mannitol or 125 mM NaCl. Bars = 0.5 cm. **(B)** Green cotyledon percentage of the wild-type, *lap2-3*, *LAP2* complementation lines and *cat2-1* mutant in panel **(A)**. **(C)** Five-days-old wild-type, *lap2-3*, *LAP2* complementation lines and *cat2-1* mutant seedlings grown on 1/2 MS medium were transferred to 1/2 MS medium without or with 250 mM mannitol or 125 mM NaCl for another 5 days, and the fresh weight was measured. Data are means (±SD) of three biological replicates. Bars with different letters in the same treatment indicate significant differences at *p* < 0.05, revealed using one-way ANOVA with Tukey’s multiple comparison test.

Previous reports showed that NAC1 and CPK3 interact with *CAT2* and positively regulate plant tolerance to salt and drought stress, leading us to further examine the role of *LAP2* in the regulation of *CAT2* activity in abiotic stresses. A previous report showed that *LAP2* mutants, *lap2-1* and *lap2-2*, are sensitive to salt and drought stresses when grown in soil, while the underlying mechanism remains largely unknown (Waditee-Sirisattha et al., 2011). To confirm these results, we identified another *LAP2* T-DNA insertion mutant, SAIL_192_A08 (named as *lap2-3*, Supplementary Figure 1A), and assayed its sensitivity to salt and osmotic stresses by adding high concentrations of NaCl or mannitol in half-strength MS medium. Consistent with previous reports, our results showed that the *lap2-3* mutant as well as *cat2-1*, the knockout mutant of *CAT2*, was indeed sensitive to high salinity and osmotic stress in terms of green cotyledon rate and changes of fresh weight (Figures 2A–C). We also generated complementation lines (*Com#1* and *Com#2*) expressing the full-length coding sequence of *LAP2* driven by its native promoter in the *lap2-3* mutant (Supplementary Figure 1B) and tested their stress sensitivity phenotype. Our results showed that the *LAP2* complementation lines recovered reduced tolerance to high salinity or osmotic stress (Figures 2A–C), verifying that the hypersensitivity of the *lap2-3* mutant to salt or osmotic stress is due to the *LAP2* gene knockout.

**FIGURE 3:**
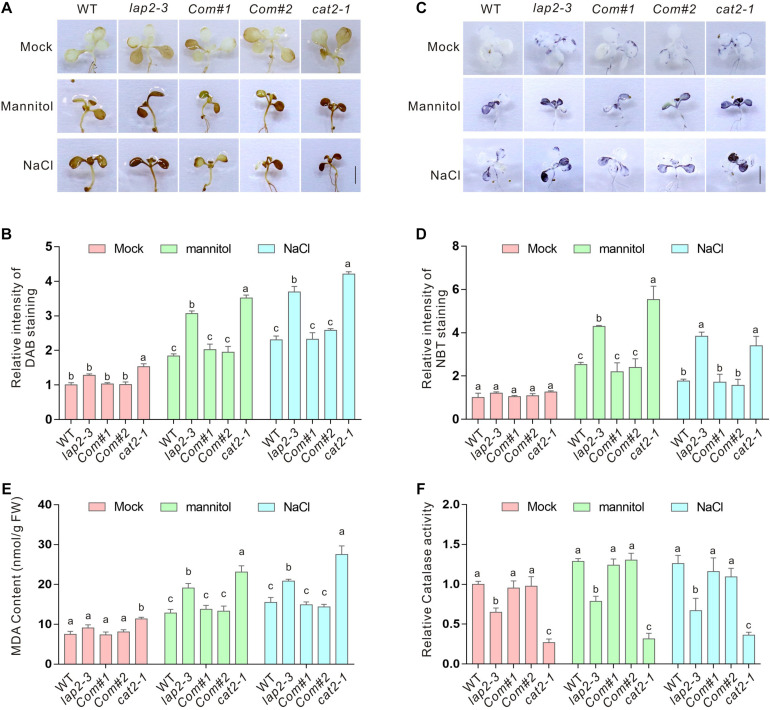
The *lap2-3* mutant has higher ROS accumulation and reduced catalase activity. **(A)** The DAB staining images of the leaves from 5-days-old wild-type, *lap2-3*, *LAP2* complementation lines and *cat2-1* mutant seedlings treated with 250 mM mannitol or 125 mM NaCl for 5 days. Bars = 0.5 cm. **(B)** The relative DAB staining intensity in panel **(A)**. The DAB staining intensity of wild-type leaves without treatment was set to 1. **(C)** The NBT staining images of the leaves from 5-days-old wild-type, *lap2-3*, *LAP2* complementation lines and *cat2-1* mutant seedlings treated with 250 mM mannitol or 125 mM NaCl for 5 days. Bars = 0.5 cm. **(D)** The relative NBT staining intensity in panel **(C)**. The NBT staining intensity of wild-type leaves without treatment was set to 1. **(E)** The MDA content of 5-days-old wild-type, *lap2-3*, *LAP2* complementation lines and *cat2-1* mutant seedlings treated with 250 mM mannitol or 125 mM NaCl for 5 days. **(F)** The catalase activities in 5-days-old wild-type, *lap2-3*, *LAP2* complementation lines and *cat2-1* mutant seedlings treated with 250 mM mannitol or 125 mM NaCl for 5 days. The catalase activity of the wild type without treatment was set to 1. Data are means (±SD) of three biological replicates. Bars with different letters in the same treatment indicate significant differences at *p* < 0.05, revealed using one-way ANOVA with Tukey’s multiple comparison test.

The interaction between *LAP2* and *CAT2* and the hypersensitivity of their mutants to salt or osmotic stress suggested an increase of ROS accumulation in the *lap2-3* mutant. As expected, significantly higher H_2_O_2_ accumulation visualized by DAB staining in the leaves was detected in both *lap2-3* and *cat2-1* mutants compared with the wild-type plants (Figures 3A,B). H_2_O_2_ overaccumulation in plants may also disturb superoxide anion metabolism. Therefore, we examined the accumulation of superoxide anion levels by NBT staining in the wild-type *lap2-3* and *cat2-1* mutant leaves and found that superoxide anion accumulation in both mutants was also substantially higher than that in the wild type (Figures 3C,D). MDA content is an indicator for the extent of cellular lipid peroxidation caused by excess ROS (Shi et al., 2012; Zhang et al., 2020a). In line with the results of the H_2_O_2_ and superoxide anion accumulation, the *lap2-3* and *cat2-1* mutants indeed had more MDA content than the wild type after treatment with high salinity or mannitol-induced osmotic stress (Figure 3E). Furthermore, we directly determined catalase activity in the wild-type and mutant plants. Our data showed that the catalase activity in the *lap2-3* and *cat2-1* mutants was significantly lower than that in the wild type either in the presence or absence of high salinity or osmotic stress treatment (Figure 3F), indicating that *LAP2* is required for catalase activity in plants under stress conditions. In addition, the *LAP2* complementation lines had similar accumulation of H_2_O_2_ and superoxide anion, MDA content, and catalase activity to the wild type (Figure 3). Together, these results revealed that *LAP2* confers plant salt or osmotic stress tolerance by promoting catalase activity and, thus, reducing ROS accumulation.

### Leucine Aminopeptidase2 Increases *CAT2* Protein Stability to Promote Plant Catalase Activity

**FIGURE 4:**
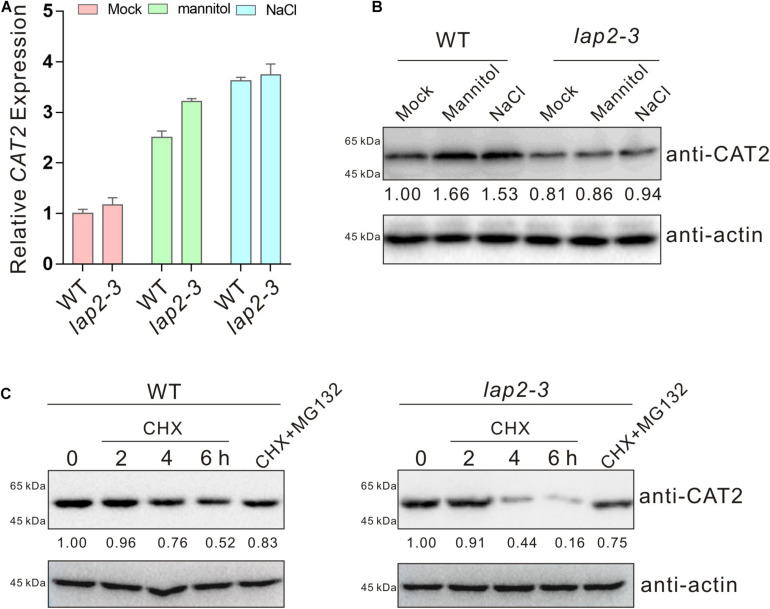
Leucine aminopeptidase2 increases *CAT2* protein stability. **(A)** The expression of *CAT2* in 5-days-old wild-type and *lap2-3* mutant seedlings treated with 250 mM mannitol or 125 mM NaCl for 5 days assayed by qRT-qPCR. The expression of *CAT2* in the untreated wild type was set as 1. Data are means (±SD) of three biological replicates. **(B)** *CAT2* protein accumulation in 5-days-old wild-type and *lap2-3* mutant seedlings treated with 250 mM mannitol or 125 mM NaCl for 5 days using anti-*CAT2* antibody. *CAT2* protein level in the untreated wild type was set as 1. Actin was used as a loading control. **(C)** Immunoblot analysis of *CAT2* protein accumulation in 5-days-old wild-type and *lap2-3* mutant seedlings incubated with 50 μM CHX for 2, 4, or 6 h or with 50 μM CHX plus 100 μM MG132 for 6 h using anti-*CAT2* antibody. *CAT2* protein level in the untreated wild type or *lap2-3* mutant was set as 1. Actin was used as a loading control.

Our above results showed a role of *LAP2* in promoting plant catalase activity, raising a possibility that *LAP2* may affect *CAT2* expression. Thus, we first assayed the transcripts of *CAT2* in the wild-type and *lap2-3* mutant seedlings treated with high concentrations of salt or mannitol using qRT-PCR. Our results showed that high salinity or osmotic stress-induced *CAT2* expression in the *lap2-3* mutant was not lower than that in the wild type (Figure 4A), excluding the possibility of *LAP2*-mediated regulation of *CAT2* transcription (Figure 4A). Next, we detected the *CAT2* protein levels in the wild-type and *lap2-3* mutant plants using anti-*CAT2* antibody which was previously reported (Zhang et al., 2020b), and our results showed that *CAT2* protein accumulation in the mutant was lower than that in the wild type either treated without or with salt or osmotic stress (Figure 4B), suggesting a role of *LAP2* in the regulation of *CAT2* protein stability. To do this, we examined the changes of *CAT2* protein accumulation in the wild-type and *lap2-3* mutant plants treated with the protein synthesis inhibitor CHX and the 26S proteasome inhibitor MG132. Our results showed that *CAT2* protein level in the *lap2-3* mutant seedlings was dramatically decreased in the presence of CHX compared with that in the wild type, while MG132 significantly represses the degradation of *CAT2* protein both in the mutant and wild-type plants (Figure 4C), revealing that *LAP2* is necessary for *CAT2* protein stability in plants.

**FIGURE 5:**
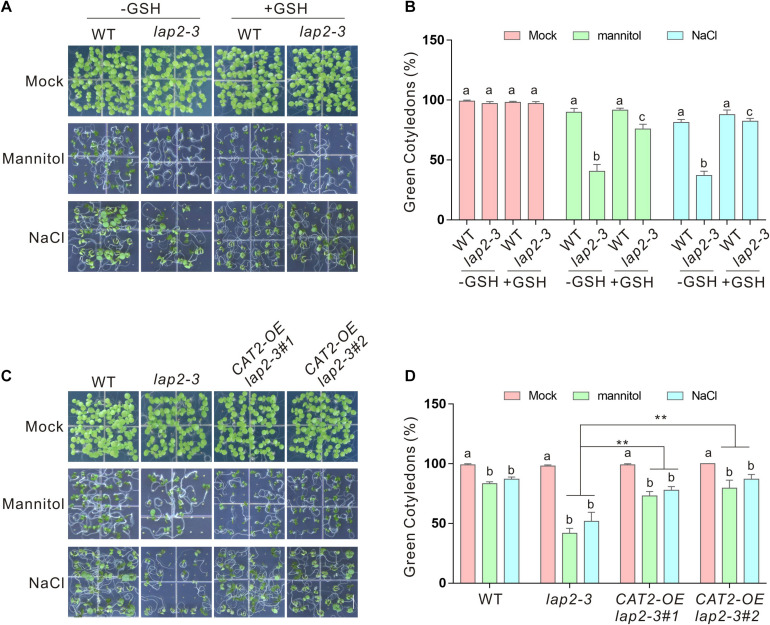
Glutathione (GSH) or overexpressing *CAT2* rescues the hypersensitivity of the *lap2-3* mutant to salt or osmotic stress. **(A)** Images of 5-days-old wild-type and *lap2-3* mutant seeds germinated on 1/2 MS medium without or with 250 mM mannitol or 125 mM NaCl in the presence or absence of 100 μM GSH. Bars = 0.5 cm. **(B)** Green cotyledon percentage of the wild type and *lap2-3* mutant in panel **(A)**. **(C)** Images of 5-days-old wild-type, *lap2-3*, and *35S:GFP-CAT2 lap2-3* (*CAT2-OE lap2-3*) transgenic plant seeds germinated on 1/2 MS medium without or with 250 mM mannitol or 125 mM NaCl. Bars = 0.5 cm. **(D)** Green cotyledon percentage of the wild-type, *lap2-3*, and *CAT2-OE lap2-3* plants in panel **(C)**. Data are means (±SD) of three biological replicates. Bars with different letters in the same treatment indicate significant differences at *p* < 0.05, revealed using one-way ANOVA with Tukey’s multiple comparison test. Asterisks indicate significant differences using Student’s *t* test (**p < 0.01).

If reduced stress tolerance of the *lap2-3* mutant was really due to ROS overaccumulation and catalase activity deficiency, scavenging the excess ROS or overexpressing *CAT2* to increase *CAT2* catalase activity should rescue the phenotype of the mutant. To test this, GSH, a widely used reducing reagent that can lower down cellular ROS accumulation, was employed (Qin et al., 2020; Zhang et al., 2020b). We found that exogenously applied GSH significantly increased the tolerance of the *lap2-3* mutant plant to salt or osmotic stress in terms of green cotyledon rate and fresh weight (Figures 5A,B). Furthermore, we generated *35S:GFP–CAT2 lap2-3* transgenic plants by overexpressing *CAT2* in the mutant (Supplementary Figure 1C) and tested their stress tolerance. Our results showed that *35S:GFP–CAT2 lap2-3* displayed a more tolerant phenotype under salt or osmotic stress conditions compared with the *lap2-3* mutant (Figures 5C,D). Together, these above results indicated that *LAP2* functions in salt- or mannitol-induced oxidative stress through increasing *CAT2* protein stability.

### *CAT2* Facilitates *LAP2* Hydrolysis Activity

**FIGURE 6:**
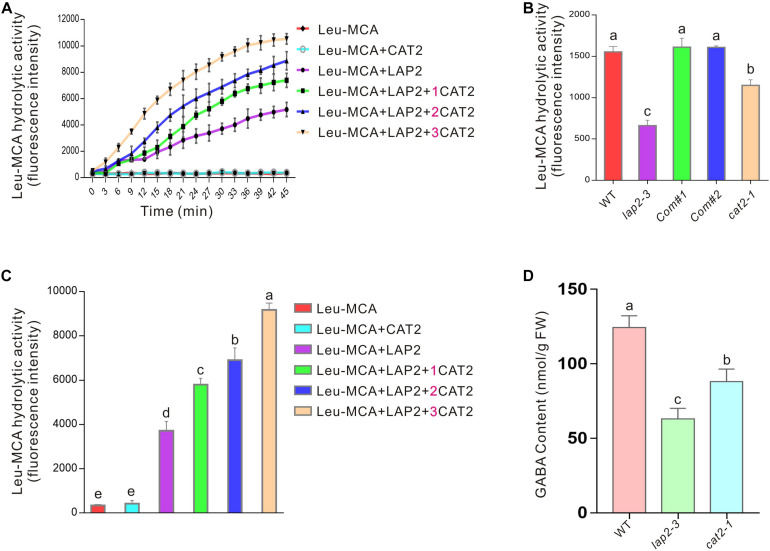
*CAT2* promotes *LAP2* activity. **(A)** The kinetics experiment of *LAP2* hydrolysis activity with Leu-MCA as the substrate in the presence or absence of *CAT2* for 45 min; 1 μg of purified *LAP2* protein and different amounts of purified *CAT2* protein (0, 1, 2, or 3 μg) were added in the reaction mixture. **(B)** The fluorescence intensities of the above experiment **(A)** at 30 min were analyzed. **(C)** The leucine peptidase activity of 10-days-old wild-type, *lap2-3*, *LAP2* complementation lines and *cat2-1* mutant seedlings with Leu-MCA as the substrate. **(D)** GABA content of 10-days-old wild-type, *lap2-3*, *LAP2* complementation lines and *cat2-1* mutant seedlings. Data are means (±SD) of three biological replicates. Bars with different letters indicate significant differences at *p* < 0.05, revealed using one-way ANOVA with Tukey’s multiple comparison test.

The above results indicate that *LAP2*, as a molecular chaperone, promotes *CAT2* protein stability so as to confer plant-enhanced salt or osmotic stress. Considering their physical interaction, we wondered whether *CAT2* affects *LAP2* activity in turn. Therefore, His-tagged *CAT2* and GST-tagged *LAP2* proteins were expressed and purified from *E. coli* BL21 (DE3), and Leu-MCA was used as a synthetic substrate of *LAP2* according to a previous report where Leu-MCA was the optimal substrate among various synthetic aminoacyl-MCAs (Waditee-Sirisattha et al., 2011). Our enzyme kinetics experiment showed that no significant changes of fluorescence signal were detected in the reaction mixture containing Leu-MCA when in the absence of *LAP2* or in the presence of *CAT2* alone, while fluorescence signal was strongly induced over time when *LAP2* was added (Figures 6A,B). Interestingly, fluorescence signal was much higher in the presence of both *CAT2* and *LAP2*, revealing that *CAT2* stimulates *LAP2* hydrolysis activity. Additionally, when more *CAT2* protein was added, fluorescence signal elicited by *LAP2* activity with Leu-MCA as the substrate was further increased (Figures 6A,B), indicating that *CAT2* promotes *LAP2* activity in a dosage-dependent manner *in vitro*.

The effect of *CAT2* on *LAP2* activity was further verified by monitoring the leucine aminopeptidase activities of the wild-type *lap2-3* and *cat2-1* mutant plants. Our results showed that both *lap2-3* and *cat2-1* harbored a lower leucine aminopeptidase activity toward the substrate Leu-MCA compared with the wild type (Figure 6C), supporting the stimulative effect of *CAT2* on *LAP2* activity *in vivo*. It is also reported that *LAP2* regulates amino acid turnover and the metabolism of a vital signaling molecule, GABA, in *Arabidopsis* (Waditee-Sirisattha et al., 2011). Thus, we further examined the GABA content in the wild-type *lap2-3* and *cat2-1* mutant plants and found that, similar to the *lap2-3* mutant, the *cat2-1* mutant also had lower GABA content than the wild type (Figure 6D).

In summary, our study revealed that *LAP2* promotes *CAT2* protein stability, while *CAT2* stimulates *LAP2* hydrolysis activity in turn, together contributing to plant tolerance to salt or osmotic stress-induced oxidative damages.

## Discussion

**FIGURE 7:**
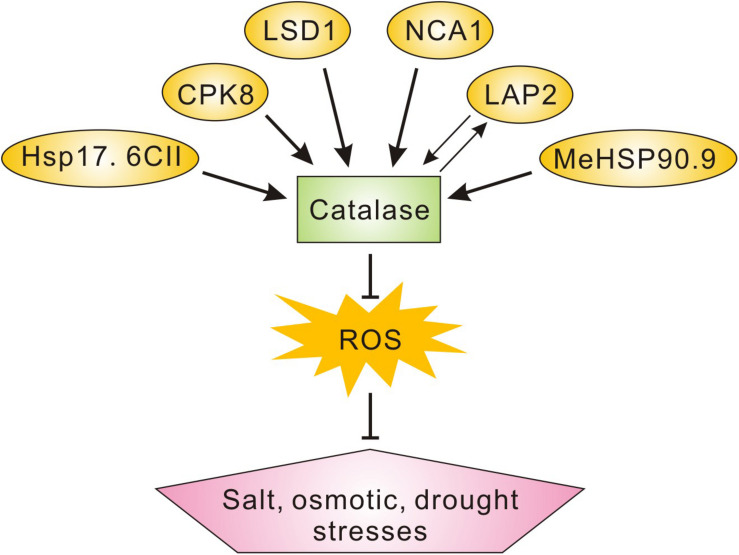
A scheme showing the catalase-interacting proteins involved in plant salt, osmotic, or drought stress tolerance. Catalase-interacting proteins including NCA1, LSD1, CPK8, MeHSP90.9, and Hsp17.6II in previous reports as well as *LAP2* in this study play roles in plant salt, osmotic, and/or drought stresses tolerance through the regulation of catalase activity and ROS homeostasis.

Catalases are ancient and conservative antioxidant enzymes across various species, catalyzing the dismutation of H_2_O_2_ to oxygen and water (Hu et al., 2010; Su et al., 2018). In contrast with other peroxidases with H_2_O_2_ as substrate, catalases do not need any reducing co-factors including ascorbate and GSH for their catalytic activity, but this may also make catalases themselves more vulnerable to excess ROS (Hu et al., 2010; Mhamdi et al., 2012; Li et al., 2015). For example, it is reported that pathogen infection-induced ROS overaccumulation results in dysfunction of catalase through direct oxidative modification (Kneeshaw et al., 2017). In this study, we identified *LAP2* as a new interacting protein of *CAT2* and also found that *LAP2* increases *CAT2* protein stability through their interaction, raising a possibility that *LAP2* may act as a molecular chaperone to protect *CAT2* from oxidative damages or maintain its structure in a functional status. Similar to this, NCA1 increases *CAT2* activity possibly through interacting with *CAT2* and facilitating its tetramer assembly (Li et al., 2015). Another *CAT2*-interacting protein, LSD1, is also required for *CAT2* activity as its loss-of-function mutant has impaired catalase activity and excess ROS (Li et al., 2013), whereas the underlying mechanism remains unclear. We have noticed that many catalase-interacting proteins have been identified including NCA1, LSD1, CPK8, MeHSP90.9, and Hsp17.6II in previous reports as well as *LAP2* in this study, and they play roles in the plant’s response and tolerance to salt, osmotic, and/or drought stresses (Figure 7). These reports unravel the mechanisms underlying the regulation of ROS homeostasis and catalase activity in plant salt or osmotic stress responses.

Our study also showed that *CAT2* could stimulate *LAP2* hydrolysis activity, affecting *LAP2*-mediated regulation of the GABA metabolism. However, we still do not know how *CAT2* affects *LAP2* activity. Previous reports showed that the peptidase activity of human dipeptidyl peptidase 8 and 9 was severely inhibited following H_2_O_2_ treatment, and moreover, reducing agents completely reversed this oxidation-mediated enzyme activity abrogation (Park et al., 2008); in addition, human dipeptidyl peptidase III could be inactivated by oxidized glutathione (GSSH), and the oxidation of the cysteine 176 participated in this enzyme inhibition (Karacic et al., 2012). These reports revealed that peptidases may be ROS-sensitive enzymes and their hydrolysis activity requires a reducing environment. In line with this, we previously also reported a similar stimulative effect of *CAT2* on acyl-CoA oxidase (ACX) 2 and ACX3 activity in the plant’s response to necrotrophic pathogen infection or during postgerminative seedling growth (Liu et al., 2017; Yuan et al., 2017). We speculated that, on the one hand, *CAT2* creates a reducing environment for these enzymes by scavenging H_2_O_2_ and, thus, protects them from oxidative damages; on the other hand, *CAT2* may affect their protein structure through interaction so as to promote their activity. These results suggest a novel role of *CAT2* in the modulation of protein activity as a regulator, in addition to its H_2_O_2_-decomposing function.

We also noticed that *LAP2* mutant leaves exhibit an early-senescence phenotype, which is similar with the *cat2-1* mutant, further supporting a tight correlation between *CAT2* and *LAP2* in plant-regulating growth and stress responses. Besides, we previously reported that jasmonic acid (JA) represses *CAT2* expression to increase H_2_O_2_ accumulation, accelerating leaf senescence (Zhang et al., 2020b). Thus, we speculated that JA-mediated repression of *CAT2* expression may also disturb *LAP2* activity, resulting in changes of metabolite flux in the leaves and, consequently, the occurrence of senescence. In addition to high salinity and osmotic stresses as we reported in this study, *LAP2* may also function in plant responses to other environmental stresses such as alkaline pH, high-intensity light, and low temperature through interacting with *CAT2*.

In summary, the results presented in this study demonstrate that *LAP2* interacts with *CAT2* and increases its protein stability, thus functioning in plant responses to ROS accumulation caused by high salinity or osmotic stress; also, *CAT2* promotes *LAP2* hydrolysis activity in turn, thus affecting GABA metabolism and plant tolerance to stresses.

## Data Availability Statement

The raw data supporting the conclusions of this article will be made available by the authors, without undue reservation.

## Author Contributions

W-CL, YZ, and L-FW designed the research. YZ, L-FW, and T-TL performed the research. YZ and L-FW analyzed the data. W-CL wrote the manuscript with contribution and approval from all authors. All authors contributed to the article and approved the submitted version.

## Conflict of Interest

The authors declare that the research was conducted in the absence of any commercial or financial relationships that could be construed as a potential conflict of interest.

## Supplementary Material

Supplementary Figure 1 Identification of *lap2-3* mutant and complementation lines. **(A)** Genomic characterization of T-DNA insertion lines of *lap2-3* (SAIL_192_A08) with the wild type as control. Primers used for PCR were listed in Supplementary Table 1. **(B)** The expression of *LAP2* in the wild-type, *lap2-3* and complementation lines were analyzed by RT-PCR. Actin was used as an internal control. Primers used for RT-PCR were listed in Supplementary Table 1. **(C)** The expression of *CAT2* in the wild-type, *lap2-3* and *35S:GFP-CAT2 lap2-3* (*CAT2-OE lap2-3*) lines were assayed by qRT-PCR. The expression of *CAT2* in the wild type was set as 1. Data are means (±SD) of three biological replicates.
