# Supplementary material for: Mutual Promotion of LAP2 and CAT2 Synergistically Regulates Plant Salt and Osmotic Stress Tolerance
Source: Front Plant Sci. 2021 Jun 9;12:672672. doi: 10.3389/fpls.2021.672672 (PMC8220078; doi:10.3389/fpls.2021.672672)
Supplement: Supplementary file 1 [file Table_1.DOCX]

**Supplemental Table S1**

List of the primers used in this study.

**Primer name Sequence (5’ to 3’)**

**Primers used for molecular cloning**

pEGAD-LAP2-F CGAGAAGCTTGGATCCATGGCCGTCACTTTGGTAACGTC

pEGAD-LAP2-R TATCTAGATCCGGTGGATTAAGAAGAAGAATGGTTCTGTACC

pEGAD-CAT2-F CGAGAAGCTTGGATCCATGGATCCTTACAAGTATCGTCC

pEGAD-CAT2-R TATCTAGATCCGGTGGATTAGATGCTTGGTCTCACGTTCAG

CAT2-Flag-F GATTACAAGGATGACGACGATAAGATGGATCCTTACAAGTATC

CAT2-Flag-R TTAGATGCTTGGTCTCACGTTCAG

LAP2pro-F  GACATGATTACGAATTCgaggatagatgagagtttgcttgctg

LAP2pro-R CCCCGGGTACCGAGCTCttgagtaacgcactgagcttctc

LAP2-F ATCCTCTAGAGTCGACATGGCCGTCACTTTGGTAACGTC

LAP2-R gaacgaaagctCTGCAGTTAAGAAGAAGAATGGTTCTGTACC

JW771-LAP2-F GacgagctcggtacccggATGGCCGTCACTTTGGTAACGTC

JW771-LAP2-R CgcgtacgagatctggtcAGAAGAAGAATGGTTCTGTACC

JW772-CAT2-F CggggcggtacccggATGGATCCTTACAAGTATCGTCC

JW772-CAT2-R Aaagctctgcaggtc-TTAGATGCTTGGTCTCACGTTCAG

YNE-LAP2-F GGCGCGCCACTAGTGGATCCATGGCCGTCACTTTGGTAACGTC

YNE-LAP2-R ACAGTACTATCGATGGATCCAGAAGAAGAATGGTTCTGTACC

YCE-CAT2-F GGCGCGCCACTAGTGGATCC-ATGGATCCTTACAAGTATCGTCC

YCE-CAT2-R ACAGTACTATCGATGGATCC-GATGCTTGGTCTCACGTTCAG

pGEX4T-LAP2-F ATCCCCGGAATTCCCGATGGCCGTCACTTTGGTAACGTC

pGEX4T-LAP2-R CGCTCGAGTCGACCCGGTTAAGAAGAAGAATGGTTCTGTACC

**Primers used for qRT-PCR or RT-PCR**

CAT2-qF tcaaaccatggatccttacaagt

CAT2-qR tgttccatacaggagcacca

LAP2-RT-F CTAGTGATAAGCTTGCAAAGGAGGT

LAP2-RT-R CTCAACTAGTGTCGCAACTCCAAAC

ACTIN2/8-F GGTAACATTGTGCTCAGTGGTGG

ACTIN2/8-R AACGACCTTAATCTTCATGCTGC

**Primers used for genomic PCR**

Lap2-3-LP CAACAACGCAAATTGTCATTG

Lap2-3-RP GATTGCTGCATCAGAGAGGAG
