# Supplementary figures and images for: Mutual Promotion of LAP2 and CAT2 Synergistically Regulates Plant Salt and Osmotic Stress Tolerance
Source: Front Plant Sci. 2021 Jun 9;12:672672. doi: 10.3389/fpls.2021.672672 (PMC8220078; doi:10.3389/fpls.2021.672672)

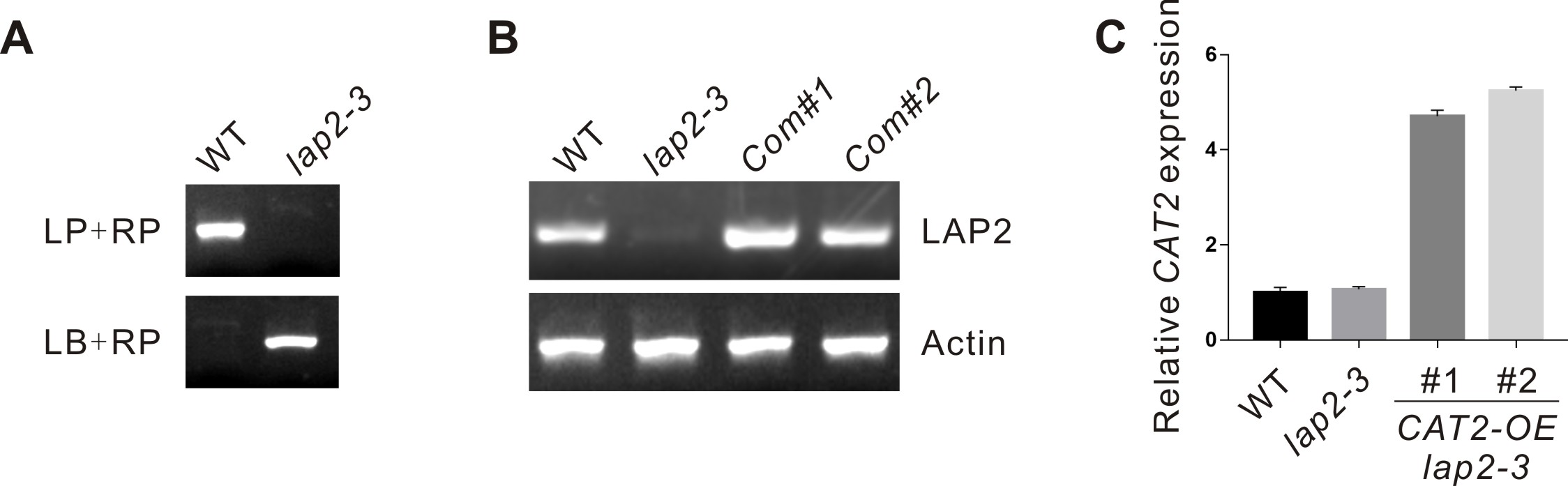

Supplement: Supplementary Figure 1 — Identification of lap2-3 mutant and complementation lines. (A) Genomic characterization of T-DNA insertion lines of lap2-3 (SAIL_192_A08) with the wild type as control. Primers used for PCR were listed in Supplementary Table 1. (B) The expression of LAP2 in the wild-type, lap2-3 and complementation lines were analyzed by RT-PCR. Actin was used as an internal control. Primers used for RT-PCR were listed in Supplementary Table 1. (C) The expression of CAT2 in the wild-type, lap2-3 and 35S:GFP-CAT2 lap2-3 (CAT2-OE lap2-3) lines were assayed by qRT-PCR. The expression of CAT2 in the wild type was set as 1. Data are means (±SD) of three biological replicates. [file Image_1.JPEG]
